# Role and perception of clinical microbiology and infectious diseases trainees during the COVID-19 crisis

**DOI:** 10.2217/fmb-2021-0129

**Published:** 2022-03-14

**Authors:** Kevin Bouiller, Nathan Peiffer-Smadja, Muge Cevik, Katharina Last, Ivana A Antunović, Anja Šterbenc, Maria J Lopes, Aleksandra Barac, Valentijn Schweitzer, Sarah Dellière

**Affiliations:** ^1^Infectious disease department, University hospital of Jean-Minjoz, Besancon, France; ^2^UMR-CNRS 6249 Chrono-environnement, Université Bourgogne Franche-Comté, Besançon, France; ^3^Network of young French infectious diseases specialists (RéJIF); ^4^Université de Paris, Service de maladies infectieuses et tropicales, Hôpital Bichat, AP-HP, Paris, France; ^5^Division of Infection & Global Health Research, School of Medecine, University of St Andrews, St Andrews, UK; ^6^Trainee association of European Society of Clinical Microbiology & Infectious Diseases (ESCMID); ^7^Center for Infectious Diseases, Institute of Medical Microbiology & Hygiene, Saarland University Hospital, Homburg, Germany; ^8^Hospital for Infections Diseases, Clinical Microbiology, Mirogojska 8, Zagreb, Croatia; ^9^Institute of Microbiology & Immunology, Faculty of Medicine, University of Ljubljana, Ljubljana, Slovenia; ^10^Infectious Diseases Department, Hospital Professor Doutor Fernando Fonseca, Amadora, Portugal; ^11^Clinic for Infectious & Tropical Diseases, University Clinical Center of Serbia, Belgrade, Serbia; ^12^Department of Medical Microbiology, University Medical Centre Utrecht, Utrecht, The Netherlands; ^13^Université de Paris, Laboratoire de Parasitologie-Mycologie, Groupe Hospitalier Saint-Louis-Lariboisière-Fernand-Widal, AP-HP, Paris, France

**Keywords:** clinical microbiology, COVID-19, infectious diseases, medical education, mental health, SARS-CoV-2, training, work–life balance, young scientists

## Abstract

**Aim:** To evaluate the role and perceptions of trainees during the COVID-19 pandemic. **Method:** An online survey was designed to provide an insight into the significance of the COVID-19 pandemic on working conditions of infectious diseases and clinical microbiology trainees. **Results:** The main roles of trainees included management of patients hospitalized for COVID-19 (55%), research (53%) and diagnostic procedures (43%). The majority (82%) of trainees felt useful in managing the crisis. However, more than two-thirds felt more stressed and more tired compared with other rotations. Only 39% of the participants had access to psychological support. **Conclusion:** Due to the significant impact of the pandemic on infectious diseases and clinical microbiology trainees, further research should focus on their health and welfare in the post-pandemic period.

The COVID-19 pandemic has caused global disruption of healthcare, forcing healthcare workers to face complex and challenging situations. This has created uncertainty particularly for postgraduate medical trainees due to the disruption of their specialist training and changes in their role, which directly impacted the completion of training requirements. The disruption of surgical care and its impact on surgical training during the COVID-19 pandemic has now been well acknowledged in the medical literature [1–5]. However, studies on the impact of the pandemic on infectious diseases (ID) and clinical microbiology (CM) trainees are sparse [6]. While all specialty trainings were affected to a certain degree, trainees in CM and ID in particular were directly involved in leading many services including the diagnosis and management of patients with COVID-19. Trainees expressed concerns about fewer learning opportunities and missing qualifying exams, and a desire to continue their training despite the pandemic [7]. In order to evaluate the role and perceptions of trainees during the COVID-19 pandemic, we conducted a survey among European ID and CM trainees with an aim to inform recommendations to fill the gaps in training and improve the well-being of those in training in the recovery phase of the pandemic.

## Materials & methods

### Study design

The survey was conceived in 2020 by the Network of Young French Infectious Disease Specialists and Steering Committee of the Trainee Association of the European Society of Clinical Microbiology and Infectious Diseases (TAE). The study included survey responses from trainees in CM and ID.

The survey was distributed online from 4 September 2020 to 6 December 2020 among CM and ID trainees in Europe using the TAE communication network, which included representatives of CM and ID national societies from 37 out of 58 (64%) European countries [8]. The survey was also promoted through the TAE website (www.escmid.org/tae), social media accounts, TAE newsletter and the European Society of Clinical Microbiology and Infectious Diseases monthly newsletters, received by more than 25,000 professionals. Although the definition of a trainee varies greatly among countries, we specifically aimed for individuals learning and practicing the skills of an ID and CM specialist.

The survey consisted of 19 multiple-choice questions and 13 questions with a five-point Likert scale (5 = strongly agree; 1 = strongly disagree), divided into four sections: demographic characteristics, role of ID and CM trainees, impact on training, and perception and feelings during the pandemic.

Before starting to fill in the questionnaire, each respondent was informed about the purpose and anonymity of the survey. No financial compensation or other incentive was provided for the respondents. Ethical approval was not required in this case due to volunteering of participants.

### Data analysis

Categorical variables were summarized by frequencies and percentages. Continuous data were presented as means with standard deviation. We compared groups using nonparametric tests for continuous variables and χ-square test for categorical variables. All analyses were performed using Excel (Microsoft Office 2016) and SPSS 22.0 (IBM Corp., NY, USA).

## Results

### Demographics

The survey was completed by 235 participants from 26 different countries. More than half were women (138/235; 59%). ID and CM trainees represented 56 and 37% of respondents, respectively, and 6% were enrolled in both training programs. Most participants were in their first, second or third year of training (23, 23 and 21%, respectively; Supplementary Table 1).

### Role in COVID-19 & impact on training

Approximately 95% of ID and CM trainees (222/235) participated in a COVID-19-related activity, of whom 34% were relocated to another medical department. The main roles of trainees included management of patients hospitalized for COVID-19 (121/222; 55%), research activities (118/235; 53%) and diagnostic procedures (96/235; 43%) (Supplementary Table 2).

The majority (193/235; 82%) of trainees felt useful in managing the crisis. However, 71% (166/235) reported working more than usual hours during the crisis, and 24% (39/166) had their working hours increased by more than 20 h per week. This was reflected in interruption in training activities. More than 90% (215/235) of respondents reported interruptions of their training, most importantly clinical training (78/215; 36%) and teaching (75/215; 35%) as well as research activities (86/215; 40%) (Supplementary Table 2).

The most challenging aspects reported were rapidly changing guidance (129/235; 55%) and distance from family and friends (108/235; 46%). The majority of the respondents (179/235; 76%) were afraid of infecting people around them, and 33% (38/235) were afraid of becoming infected with SARS-CoV-2. Infection control measures were considered insufficient by one-quarter of respondents (54/235).

### Perception & feelings during the pandemic

**Figure 1.**
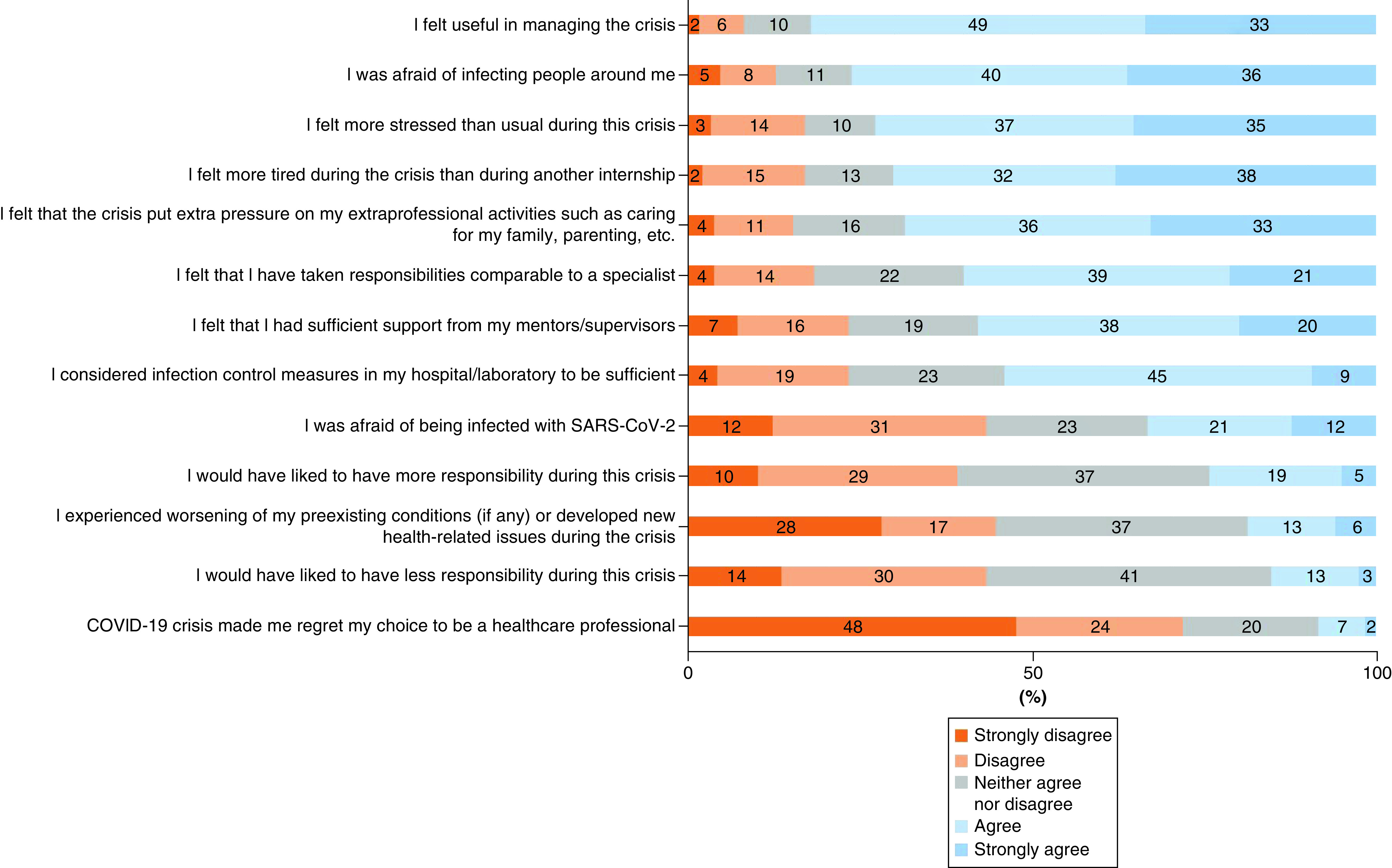
Impact of COVID-19 on infectious diseases and clinical microbiology trainees (Likert scale).

More than two-thirds of trainees felt more stressed and more tired compared with other training courses during internships (Figure 1). Moreover, 19% (45/235) of the participants experienced worsening of their pre-existing medical conditions. Only 39% (91/235) of the participants had access to psychological support (Figure 1). Among the trainees who were unwell (including with COVID-19), 75% (49/65) benefited from a statutory sick leave.

Overall, financial compensation was offered to 63% of trainees (147/235), with significant differences among countries. More than two-thirds of trainees from Sweden (6/9; 67%), Germany (6/9; 67%), Great Britain (18/23; 78%), France (59/63; 94%) and Slovenia (15/15; 100%) were paid for extra hours and/or received a bonus. On the contrary, fewer trainees from Belgium (4/21; 19%), Spain (4/14; 29%), Italy (3/9; 33%), Portugal (4/8; 50%) and Croatia (7/13; 54%) received financial compensation.

### Differences between countries

There were significant differences between European countries. Trainees in southern and eastern Europe were more likely to relocate to another department compared with those in western and northern European countries (42 vs 29%; p = 0.048; Supplementary Table 3). Similarly, they felt more stressed (89 vs 75%; p = 0.026), more tired (88 vs 76%; p = 0.042) and would have liked to have less responsibility during the crisis (38 vs 19%; p = 0.018). They were more afraid of being infected with SARS-CoV-2 (64 vs 32%; p < 0.001). Finally, a greater number of trainees from southern and eastern Europe felt less useful in managing the crisis (16 vs 7%; p = 0.037) (Supplementary Table 4).

## Discussion

The results of this study demonstrate that while most ID/CM trainees felt helpful in managing the crisis, they reported an increased workload with irregular shift patterns, a substantial negative impact on psychological health, experiencing increasing stress and exhaustion, and major disruption in training activities. Most worryingly, less than two-thirds of trainees were offered financial compensation for the additional workload, which was far lower in some countries than others, and less than half had access to support services.

Service priorities and safety considerations during the COVID-19 pandemic disrupted training for all medical trainees, including those in anesthesiology and intensive care [5]. In particular, the increased workload had negative consequences for teaching and training, which is a significant cause of concern. In addition, changes related to management of COVID-19 have had consequences on trainees’ well-being and mental health [5,9]. Other medical trainees identified working in personal protective equipment and fear of becoming infected or infecting their colleagues as causes for concern [2]. The COVID-19 pandemic was clearly identified as a cause of psychological distress for all healthcare providers [10]. For instance, during COVID-19, far more otolaryngology residents experienced burnout than attending physicians, probably because of increased work hours and the nature of their work [11]. In this analysis, more than two-thirds of trainees felt more stressed and more tired compared with other training courses during internships, Therefore it is important to focus our efforts on ensuring that trainees have adequate time off to prevent physical and emotional exhaustion during crises. In addition, post-pandemic recovery plans need to include psychological support and care which is essential for the well-being of trainees [12].

Interestingly, we found some discerning characteristics among southern and eastern European countries. However, with respect to the large number of variables analyzed, different training schemes and various countries included, further studies focusing on individual variables should be performed to confirm these results.

One of the positive consequences of the COVID-19 pandemic was the opportunity for trainees to be involved in various research activities. Indeed, enthusiasm for research was identified, as 53% of trainees reported participation in research activities. However, this survey did not evaluate whether this involvement was voluntary or mandatory.

Some specific aspects of CM and ID trainees’ work during the pandemic may have had a big impact on their training:

- ID departments were often integrally used for COVID-19 patients, which decreased the management of other IDs and reduced the spectrum of training. Similarly, non-COVID-19-related activities in CM laboratories were halted or significantly reduced.
- Senior doctors and mentors were occupied by their responsibilities in managing the crisis, which may have led to a decrease in the quality of mentorships and supervision.
- Higher expectations from their medical peers in being up to date with the literature regarding the pathophysiology, diagnosis and management of COVID-19 patients.

The results of this study may not be representative of all CM and ID trainees in Europe, as several large European countries (e.g., Germany, Italy and Turkey) were clearly under-represented [8]. Another limitation is the difficulty of estimating the response percentage of this survey, given that the survey was promoted through the TAE website, social media accounts and TAE newsletters, which did not allow for an accurate assessment of the number of potential responders.

## Conclusion

The COVID-19 pandemic has led to a considerable change in training, with uncertain effects on ongoing specialty training for many. Given the significant consequences of the COVID-19 pandemic both on training and on the psychological needs of trainees as shown in this analysis, measures should be taken to ensure continuity of training needs. In addition, appropriate financial compensation and systematic psychological support should be considered part of the pandemic management rather than an afterthought. Much more importance should be placed on the training, health and well-being needs of trainees during the recovery phase of the crisis.

**Summary points**

- The majority of infectious disease and clinical microbiology trainees actively participated in clinical and research activities during the COVID-19-pandemic.
- 82% of trainees felt helpful in managing the crisis.
- Negative points of the pandemic were: increased workload, irregular shift patterns, more stress than usual, interruption of training.
- Many respondents reported changes in psychological state, but there was a lack of systematically provided psychological care.
- The specific impacts on trainees in different European countries should be evaluated in further studies.
- The consequences of the pandemic for specialty recruitment should be evaluated after the crisis.
