# Supplementary material for: Role and perception of clinical microbiology and infectious diseases trainees during the COVID-19 crisis
Source: Future Microbiol. 2022 Mar 14:10.2217/fmb-2021-0129. doi: 10.2217/fmb-2021-0129 (PMC8958984; doi:10.2217/fmb-2021-0129)
Supplement: Supplementary file 1 [file Supplementary_data.docx]

|  |  | Total (n=235) |
| --- | --- | --- |
| Sex, n (%) | Female | 138 (59) |
|  | Male | 96 (41) |
|  | Non-binary | 1 (0.43) |
| Age, mean (± SD) |  | 31.3 (6.01) |
| Country | France  Great Britain  Belgium  Austria  Slovenia  Spain  Croatia  Sweden  Germany  Italy  Portugal  Greece  Denmark  Turkey  Switzerland  Bulgaria  Romania  Serbia  Ukraine  Luxembourg  Estonia  Non-European country* | 63 (26.8)  23 (9.8)  21 (8.9)  15 (6.4)  15 (6.4)  14 (6.0)  13 (5.5)  9 (3.8)  9 (3.8)  9 (3.8)  8 (3.4)  5 (2.1)  4 (1.7)  4 (1.7)  3 (1.3)  2 (0.9)  2 (0.9)  2 (0.9)  1 (0.4)  1 (0.4)  1 (0.4)  11 (4.7) |
| Training program, n (%) | Infectious diseases (ID) | 132 (56.2) |
|  | Clinical microbiology (CM) | 88 (37.4) |
|  | Both (ID and CM) | 15 (6.4) |
| Years of training completed, n (%) | 1 | 53 (22.6) |
|  | 2 | 54 (23.0) |
|  | 3 | 50 (21.3) |
|  | 4 | 34 (14.5) |
|  | 5 | 15 (6.4) |
|  | >5 | 29 (12.3) |

Supplementary table 1. Demographics data of trainees who completed the survey

India (n=5), Chile (n=3), USA (n=1), Canada (n=1), Republic of Korea (n=1)

Supplementary table 2. Answers to the survey of 235 trainees in clinical microbiology (CM) and infectious diseases (ID)

|  |  | TOTAL (n=235) |
| --- | --- | --- |
| Participation in COVID-19 activity | YES | 222 (94.5) |
| What activities? | Hospitalization | 121 (54.5) |
|  | Research activities | 118 (53.2) |
|  | Diagnostic procedure (swab) | 96 (43.2) |
|  | Laboratory (PCR) | 63 (28.4) |
|  | Writing guidelines/protocols | 60 (27.0) |
|  | Diagnostic consultation | 59 (26.6) |
|  | Follow-up consultation (phone) | 52 (23.4) |
|  | ICU | 51 (23.0) |
|  | Follow-up consultation (face to face) | 39 (17.6) |
|  | Contact tracing | 29 (13.1) |
| Relocate to another department | YES | 79 (33.6) |
| What department? | ID department | 37 (46.8) |
|  | COVID laboratory | 17 (21.5) |
|  | COVID department (non-ID dpt) | 16 (20.2) |
|  | ICU | 8 (10.1) |
|  | Infection control | 6 (7.6) |
|  | Public health department | 3 (3.8) |
|  | Research department | 3 (3.8) |
|  | Other | 2 (2.5) |
| Financial compensation | Yes | 147 (62.6) |
| Which one? | Bonus | 67 (45.6) |
|  | Paid for extra hours | 56 (38.1) |
|  | Both | 24 (16.3) |
| Interruption of training | YES | 215 (91) |
| What activities? | Conference/congresses | 132 (61) |
|  | Research | 86 (40) |
|  | Clinical/lab rotation | 79 (37) |
|  | Clinical activity in a department | 78 (36) |
|  | Teaching | 75 (35) |
|  | University training | 61 (28) |
|  | Clinical consultation | 49 (23) |
|  | Laboratory | 41 (19) |
|  | PhD/MSc work | 33 (15) |
| Work more than usually | Yes | 166 (70.6) |
| How many hours/week more | 0-5 | 38 (22.9) |
|  | 5-10 | 53 (31.9) |
|  | 10-20 | 36 (21.7) |
|  | >20 | 39 (23.5) |
| Benefit from a sick leave if unwell | Yes | 49/65 (75.4) |
| Possibility of psychological support | Yes | 91 (38.7) |
| Most difficult things to manage | Rapidly changing guidance | 129 (54.9) |
|  | Distance from family/friends | 108 (46.0) |
|  | Lack of communication | 73 (31.1) |
|  | Lack of data on COVID-19 | 72 (30.6) |
|  | Lack of guidance | 72 (30.6) |
|  | Working in PPE | 51 (21.7) |
|  | Keeping up with emerging evidence | 50 (21.3) |
|  | Different shifts (night work) | 48 (20.4) |
|  | Abundance of literature | 45 (19.2) |

ID : infectious disease, MSc : Master of science, ICU : intensive care unit, dpt : department, PPE: personal protective equipment

Supplementary table 3. Characteristics of participants between Southern/Eastern and other European countries

|  | Other European countries** (n=149) | Southern and Eastern European countries* (n=75) | p |
| --- | --- | --- | --- |
| Age, mean ±SD | 31.4 (±6.85) | 30.8 (±4.10) | 0.39 |
| Sex, n (%) | | | |
| Female | 85 (57) | 51 (68) | 0.11 |
| Male | 64 (43) | 23 (31) |  |
| Non-binary | 0 | 1 (1) |  |
| Training programm | | | |
| Infectious diseases (ID) | 93 (62) | 33 (44) | <0.001 |
| Clinical microbiology (CM) | 44 (30) | 40 (53) |  |
| Both (ID and CM) | 12 (8) | 2 (3) |  |
| Years of training, n | | | |
| 1 | 35 (23) | 15 (20) | 0.41 |
| 2 | 28 (19) | 22 (29) |  |
| 3 | 35 (23) | 15 (20) |  |
| 4 | 20 (13) | 12 (16) |  |
| 5 | 9 (6) | 5 (6.7) |  |
| >5 | 22 (15) | 6 (8) |  |
| Participation in COVID-19 activity | 142 (95) | 69 (92) | 0.37 |
| Hospitalization | 88 (59) | 26 (35) | <0.01 |
| Research activities | 69 (46) | 41 (55) | 0.24 |
| Diagnostic procedure (swab) | 60 (40) | 31 (41) | 0.88 |
| Laboratory (PCR) | 32 (21) | 27 (36) | 0.02 |
| Writing guidelines/protocols | 35 (23) | 18 (24) | 0.93 |
| Diagnostic consultation | 35 (23) | 19 (25) | 0.76 |
| Follow-up consultation (phone) | 35 (23) | 19 (25) | 0.76 |
| ICU | 40 (27) | 6 (8) | <0.01 |
| Follow-up consultation (face to face) | 25 (17) | 9 (12) | 0.35 |
| Contact tracing | 20 (13) | 8 (11) | 0.56 |
| Relocate to another department | 43 (29) | 31 (41) | 0.048 |
| Financial compensation | 98 (66) | 43 (57) | 0.22 |
| Interruption of training | 133 (89) | 71 (95) | 0.18 |
| Conference/congresses | 77 (52) | 48 (64) | 0.08 |
| Clinical activity in a department | 46 (31) | 28 (37) | 0.33 |
| University training | 44 (30) | 11 (15) | 0.018 |
| Research | 53 (36) | 29 (39) | 0.65 |
| Teaching | 50 (34) | 17 (23) | 0.093 |
| Clinical consultation | 38 (26) | 9 (12) | 0.019 |
| Clinical/lab rotation | 30 (20) | 42 (56) | <0.01 |
| Laboratory | 22 (15) | 14 (19) | 0.42 |
| PhD/MSc work | 23 (15) | 10 (13) | 0.68 |
| Work more than usually | 101 (68) | 56 (75) | 0.29 |
| >20 hours/week more | 20 (13) | 14 (19) | 0.3 |
| Benefit from a sick leave if unwell | 33 (72) | 9 (75) | 1 |
| Possibility of psychological support | 58 (39) | 26 (35) | 0.53 |
| Most difficult things to manage | | | |
| Rapidly changing guidance | 95 (64) | 30 (40) | <0.01 |
| Distance from family/friends | 74 (50) | 31 (41) | 0.24 |
| Lack of communication | 40 (27) | 31 (41) | 0.028 |
| Lack of data on COVID-19 | 42 (28) | 27 (36) | 0.23 |
| Lack of guidance | 37 (25) | 33 (44) | <0.01 |
| Working in PPE | 27 (18) | 18 (24) | 0.3 |
| Keeping up with emerging evidence | 40 (27) | 7 (9.3) | <0.01 |
| Different shifts (night work) | 26 (17) | 20 (27) | 0.11 |
| Abundance of literature | 37 (25) | 5 (6.8) | <0.01 |

* Slovenia, Serbia, Greece, Portugal, Italy, Spain, Turkey, Croatia, Ukraine, Romania, Bulgaria

** Non-European countries were excluded: India (n=5), Chile (n=3), USA (n=1), Canada (n=1), Republic of Korea (n=1)

Supplementary table 4. Comparison of responses between South-Eastern European countries to other European countries

|  | Other European countries** (n=149) | South-Eastern European countries* (n=75) | p |
| --- | --- | --- | --- |
| I felt useful in managing the crisis ⱡ | 131 (95%) | 52 (81%) | <0.01 |
| I felt more stressed than usual during the crisis ⱡ | 98 (75%) | 62 (89%) | 0.026 |
| I felt more tired during the crisis than during another internship ⱡ | 97 (76%) | 61 (88%) | 0.042 |
| I was afraid of becoming infected with SARS-CoV-2 ⱡ | 37 (32%) | 35 (64%) | <0.001 |
| I was afraid of infecting people around me ⱡ | 108 (83%) | 61 (88%) | 0.32 |
| I felt that crisis put extra pressure on my various tasks such as family, parenting, caring for sick/elderly family members ⱡ | 98 (80%) | 52 (81%) | 0.88 |
| I thought that infection control measures in my hospital/laboratory were sufficient. ⱡ | 83 (72%) | 39 (68%) | 0.61 |
| I would have liked to have had more responsibility during this crisis ⱡ | 31 (36%) | 22 (40%) | 0.67 |
| I would have liked to have had less responsibility during this crisis ⱡ | 16 (19%) | 18 (38%) | 0.018 |
| I felt that I have taken responsibility comparable to a specialist ⱡ | 86 (78%) | 46 (71%) | 0.27 |
| I felt that I had sufficient support from my mentors/supervisors ⱡ | 94 (75%) | 36 (64%) | 0.13 |
| I did experienced worsening of my preexisting conditions (if you have any) or developed new health-related issues during the crisis ⱡ | 27 (31%) | 16 (29%) | 0.79 |
| COVID-19 crisis makes me regret to have chosen to be a health care professional ⱡ | 8 (6.5%) | 9 (16%) | 0.037 |

ⱡ Only responses “agree” or “strongly agree” were retained in the analysis
